# Detection of hepatocarcinogens by combination of liver micronucleus assay and histopathological examination in 2-week or 4-week repeated dose studies

**DOI:** 10.1186/s41021-021-00222-1

**Published:** 2022-01-04

**Authors:** Shuichi Hamada, Miyuki Shigano, Yumi Wako, Kazufumi Kawasako, Kensuke Satomoto, Tatsuya Mitsumoto, Takayuki Fukuda, Wakako Ohyama, Takeshi Morita, Makoto Hayashi

**Affiliations:** 1 BoZo Research Center Inc, 1-3-11 Hanegi, Setagaya-ku, Tokyo, 156-0042 Japan; 2 LSIM Safety Institute Corporation, 14-1 Sunayama, Kamisu-shi, Ibaraki, 314-0255 Japan; 3 Rakuno Gakuen University, 582 midorimachi, Bunkyoudai, Ebetsu-shi, Hokkaido 069-8501 Japan; 4 Yakult Honsha Co., Ltd, 5-11 Izumi, Kunitachi-shi, Tokyo, 186-8650 Japan; 5 National Institute of Technology and Evaluation, 2-49-10 Nishihara, Shibuya-ku, Tokyo, 151-0066 Japan; 6 makoto international consulting, 4-23-3-1 Kamiimaizumi, Ebina-shi, Kanagawa 243-0431 Japan

**Keywords:** Micronucleus assay, Liver, Hepatocarcinogen, Histopathology, Early responses

## Abstract

**Background:**

Currently, revisions to the ICH S1 guidance on rodent carcinogenicity testing are being proposed. Application of this approach would reduce the use of animals in accordance with the 3Rs principles (reduce/refine/replace). The method would also shift resources to focus on more scientific mechanism-based carcinogenicity assessments and promote safe and ethical development of new small molecule pharmaceuticals. In the revised draft, findings such as cellular hypertrophy, diffuse and/or focal cellular hyperplasia, persistent tissue injury and/or chronic inflammation, preneoplastic changes, and tumors are listed as histopathology findings of particular interest for identifying carcinogenic potential. In order to predict hepatocarcinogenicity of test chemicals based on the results from 2- or 4-week repeated dose studies, we retrospectively reanalyzed the results of a previous collaborative study on the liver micronucleus assay. We focused on liver micronucleus induction in combination with histopathological changes including hypertrophy, proliferation of oval cells or bile duct epithelial cells, tissue injuries, regenerative changes, and inflammatory changes as the early responses of hepatocarcinogenesis. For these early responses, A total of 20 carcinogens, including 14 genotoxic hepatocarcinogens (Group A) and 6 non-liver-targeted genotoxic carcinogens (Group B) were evaluated.

**Results:**

In the Group A chemicals, 5 chemicals (NPYR, MDA, NDPA, 2,6-DNT, and NMOR) showed all of the 6 early responses in hepatocarcinogenesis. Five chemicals (DMN, 2,4-DNT, QUN, 2-AAF, and TAA) showed 4 responses, and 4 chemicals (DAB, 2-NP, MCT, and Sudan I) showed 3 responses. All chemicals exhibited at least 3 early responses.

Contrarily, in the Group B chemicals (6 chemicals), 3 of the 6 early responses were observed in 1 chemical (MNNG). No more than two responses were observed in 3 chemicals (MMC, MMS, and KA), and no responses were observed in 2 chemicals (CP and KBrO3).

**Conclusion:**

Evaluation of liver micronucleus induction in combination with histopathological examination is useful for detecting hepatocarcinogens. This assay takes much less time than routine long-term carcinogenicity studies.

## Introduction

The liver is an important tissue not only in general toxicological studies, but also in carcinogenicity studies. About 60% of carcinogens are hepatocarcinogens [1], suggesting that development a new evaluation method targeting the liver is meaningful. In addition to the routinely used erythropoietic micronucleus in rodents, the liver micronucleus assay has been developed to detect genotoxic hepatocarcinogens that require metabolic activation [2–6].

The liver micronucleus assay targets the primary organ for drug metabolism; however, it is not commonly used due to slow hepatocyte proliferation in adult rats. Partial hepatectomy [7–9], mitogen treatment [10, 11], and the use of juvenile rats [12–15] have been introduced to address this drawback. Unfortunately, these methods have disadvantages, including complex surgical procedures and decreased metabolic activity for partial hepatectomy [16], risk of drug interactions for mitogen treatment [17], and a lack of maturation for metabolic activation in juvenile rats [18]. Recently, a repeated-dose liver micronucleus assay (RDLMN) was developed as a new method for evaluating liver micronuclei. The approach used 2- or 4-week repeated-dose treatment for the accumulation of micronucleated hepatocytes (MNHEPs) [19]. This method facilitates the integration of the liver micronucleus assay into repeated-dose general toxicity studies to simultaneously assess genotoxicity and histopathological endpoints with the same animals used for the overall evaluation of chemical risk.

Routine long-term carcinogenicity studies are time consuming and costly and require large numbers of animals. Revision to the ICH S1 guidelines is being discussed to address these issues. In a revised draft, histopathological findings such as cellular hypertrophy, diffuse and/or focal cellular hyperplasia, persistent tissue injury and/or chronic inflammation, preneoplastic changes, and tumors are listed as particular interest for identifying carcinogenic potential [20]. The possibility of predicting hepatocarcinogenicity of test chemicals based on the results of 2- or 4-week repeated-dose studies was assessed using a reanalysis of a previous collaborative study of the liver micronucleus assay [2, 21] in combination with histopathological examination.

## Materials and methods

### Classification of chemicals and previous collaborative study by CSGMT/JEMS MMS

Twenty genotoxic carcinogens examined in a previous collaborative study by CSGMT/JEMS MMS were classified into two groups: Group A consisted of 14 genotoxic hepatocarcinogens and Group B consisted of 6 non-liver-targeted genotoxic carcinogens. Liver micronucleus assay data were then integrated (Table 1).

**Table 1:** Liver MN assay results in the collaborative study by CSGMT/JEMS MMS and rat carcinogenicity data for the test chemicals

Group	Chemical	Abbreviation	CAS no.	In vivo MN assay (Liver)				Rat carcinogenicity		
				2 weeks	Ref.	4 weeks	Ref.	Liver	Other sites	Ref.
Group A	Dimethylnitrosamine	DMN	62–75-9	+	[2]	+	[2]	+	kid, lun, vsc, tes	[22]
	*N*-Nitrosopyrrolidine	NPYR	930–55-2	+	[2]	+	[2]	+	kid, vsc, tes	[22, 23]
	4,4′-Methylenedianiline	MDA	101–77-9	+	[2]	+	[2]	+	thy	[24]
	*N*-Nitrosodipropylamine	NDPA	621–64-7	+	[2]	ND		+	eso, nas	[22]
	2,4-Dinitrotoluene	2,4-DNT	121–14-2	+	[2]	+	[2]	+	ski, mgl	[22]
	2,6-Dinitrotoluene	2,6-DNT	606–20-2	+	[2]	+	[2]	+	–	[22]
	Quinoline	QUN	91–22-5	+	[2]	+	[2]	+	–	[25]
	*p*-Dimethylaminoazobenzene	DAB	60–11-7	+	[2]	+	[2]	+	–	[22]
	2-Nitropropane	2-NP	79–46-9	+	[2]	+	[2]	+	–	[26]
	Monocrotaline	MCT	315–22-0	+	[2]	+	[2]	+	–	[22]
	*N*-Nitrosomorpholine	NMOR	59–89-2	+	[2]	ND		+	vsc	[22]
	2-Acetylaminofluorene	2-AAF	53–96-3	+	[2]	+	[2]	+	ski, mgl	[22]
	Sudan I (C.I.solvent yellow 14)	Sudan I	842–07-9	+	[21]	ND		+	–	[22]
	Thioacetamide	TAA	62–55-5	+	[21]	+	[21]	+	–	[22]
Group B	Mitomycin C	MMC	50–07-7	+	[2]	+	[2]	–	per	[22]
	Cyclophosphamide H2O	CP	6055-19-2	–	[2]	ND		–	ub, lym, ner	[27]
	Potassium bromate	KBrO3	7758-01-2	–	[2]	–	[2]	–	kid, per, thy	[22]
	*N*-Methyl-*N*′-nitro-*N*-nitrosoguanidine	MNNG	70–25-7	–	[2]	–	[2]	–	eso, smi, sto	[22]
	Methyl methanesulfonate	MMS	66–27-3	+	[2]	–	[2]	–	hmo, lun, ner	[22, 26]
	Kojic acid	KA	501–30-4	–	[2]	–	[2]	–	thy (mouse)	[22]

MN assay: micronucleus assay

+: positive; −: negative; ND: no data;

kid: kidney; lun: lung; vsc: vascular system; tes: testes; thy: thyroid gland; eso: esophagus; nas: nasal cavity; ski: skin; mgl: mammary gland; per: peritoneal cavity;

ub: urinary bladder; lym: lymphocyte; ner: nervous system; smi: small intestine; sto: stomach; hmo: hematopoietic system; pan: pancreas

Group A, Genotoxic hepatocarcinogens;

Group B, Genotoxic carcinogens but non-liver-targeted

Male Crl:CD (SD) rats used in the previous report [28–44] were purchased from Charles River Japan Inc. (Atsugi, Hino or Tsukuba, Japan) and used at the age of 6 weeks. The animals were housed in an air-conditioned room with a 12-h light/dark cycle and allowed free access to food and water. The animal experiments were approved by the Institutional Animal Care and Use Committee of each testing facility in advance. The rats (5/group) were given each chemical repeatedly by oral gavage for 14 or 28 consecutive days. Twenty-four hours after the last administration, the rats were euthanized under thiopental anesthesia. Livers were removed and a part of each liver (left lateral lobe) was used for the liver micronucleus assay [28–44]. The remaining tissue was fixed with 10% phosphate-buffered formalin, embedded in paraffin, thin-sectioned, and stained with hematoxylin and eosin according to standard protocols. Histopathological examination was performed by a pathologist using light microscopy.

### Reanalysis of pathological findings and application to the hepatocarcinogenesis process

**Fig. 1:**
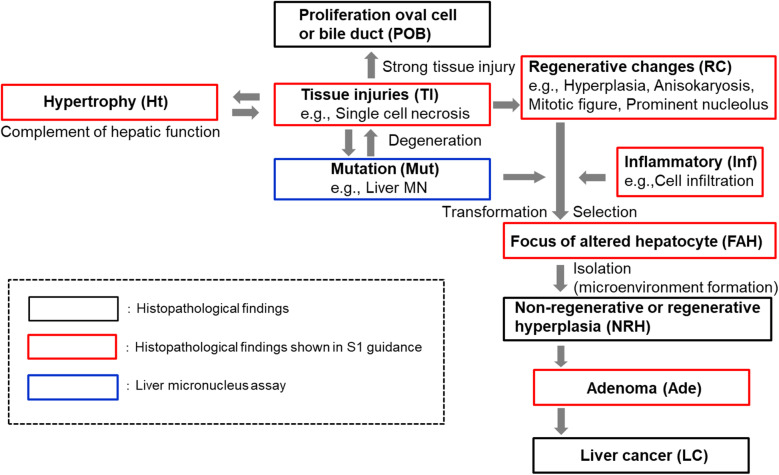
Processes in multistage carcinogenesis theory and pathological findings in proposed changes to ICH S1 guidance

Common markers for a precancerous stage in hepatocarcinogenesis include (i) transformation of normal hepatocytes into preneoplastic hepatocytes, (ii) selection of preneoplastic hepatocytes for growth, and (iii) isolation of preneoplastic hepatocytes from normal hepatic tissue. Transformation, selection, and isolation are thus general processes for the progression of preneoplastic hepatocytes into malignant cells [45]. With references to this report and the histopathology findings of particular interest for identifying carcinogenic potential pointed out in the draft S1 guidelines [20], changes in each carcinogenic process were roughly divided into 10 categories: mutation (including liver micronucleus induction), hypertrophy, tissue injuries, proliferation of oval cells or bile duct epithelial cells, regenerative changes, inflammatory changes, focus of altered hepatocytes, non-regenerative or regenerative hyperplasia, adenoma, and liver cancer (Fig.1).

We used the above information to reanalyze the presence of 9 liver pathological responses based on the findings from the previous collaborative study. Each of the 20 chemicals was reassessed. The grades of findings and frequency of appearance were disregarded to simplify the evaluation. Judgment was used only for the presence or absence determination. Except for accidental findings, findings judged to result from toxic insult were comprehensively evaluated. Mutation was identified via induction of liver micronuclei. Chemicals evaluated in 14- and 28-day repeated dose studies were judged to be “with findings” if chemical-related toxicity was observed in either time frame. Chemicals without findings in either time frame were judged to be “without findings”.

## Results

### Group A chemicals (genotoxic hepatocarcinogens)

**Fig. 2:**
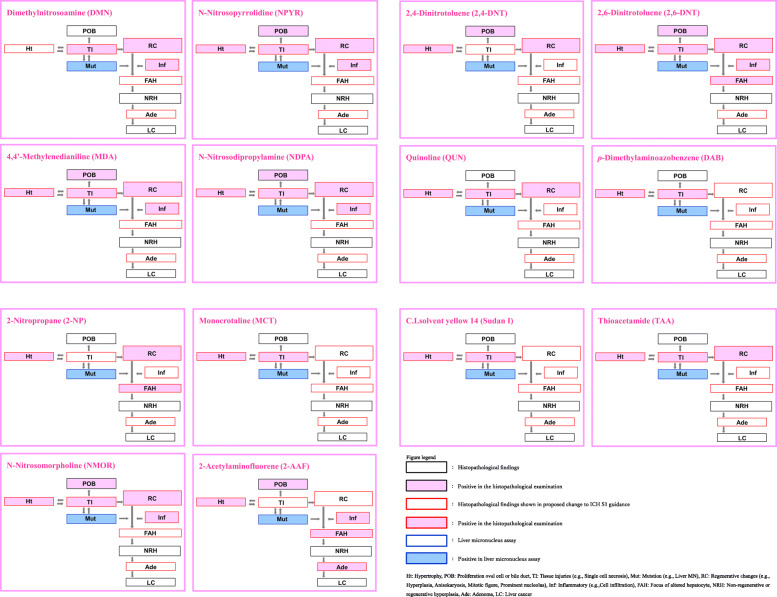
Liver micronucleus induction and histopathological changes observed in 14-day and/or 28-day repeated-dose studies – genotoxic hepatocarcinogens

We evaluated 14 Group A chemicals for 10 markers of the carcinogenic pathways (9 liver pathological responses and liver micronucleus induction) (Fig.2). The liver micronucleus induction was most frequently observed (100% [14/14]) followed by hypertrophy (93% [13/14]), tissue injuries (79% [11/14]), proliferation of oval cells or bile duct epithelial cells (50% [7/14]), regenerative changes (71% [10/14]), inflammatory changes (50% [7/14]), focus of altered hepatocytes (21% [3/14]), and adenomas (7% [1/14]). Non-regenerative or regenerative hyperplasia and liver cancer were not observed.

One chemical (2,6-DNT) demonstrated 7 of the 10 aforementioned responses. Five chemicals (NPYR, MDA, NDPA, NMOR, and 2-AAF) displayed 6 responses, 5 chemicals (DMN, 2,4-DNT, QUN, 2-NP, and TAA) exhibited 4 responses, and 3 chemicals (DAB, MCT, and Sudan I) showed 3 responses. No chemical showed fewer than three responses.

### Group B chemicals (genotoxic carcinogens but not liver targeted)

**Fig. 3:**
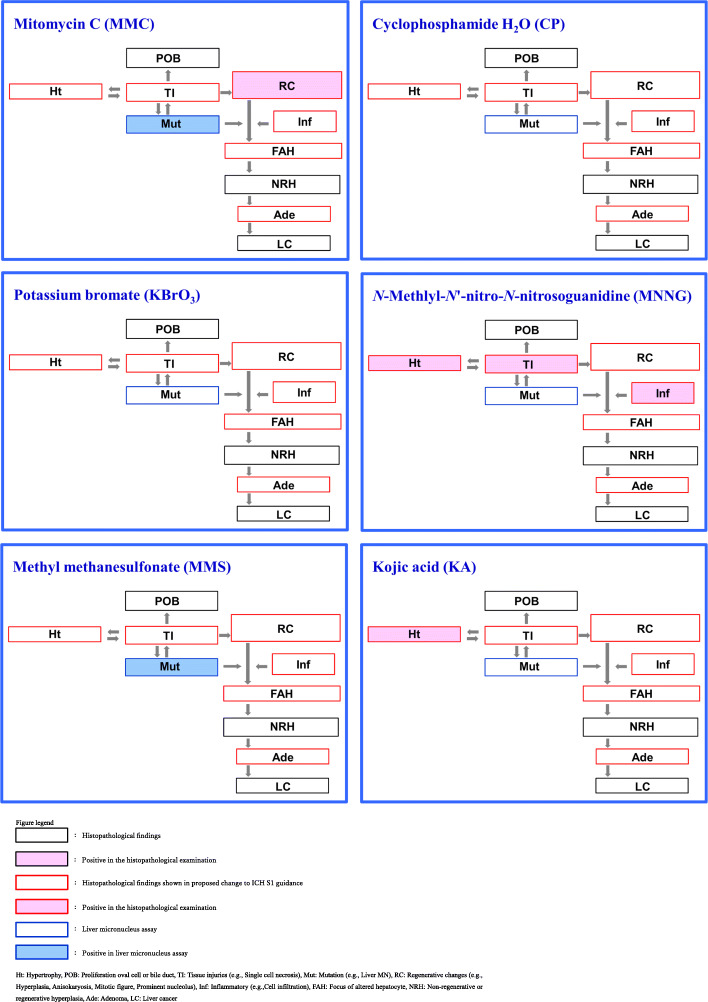
Liver micronucleus induction and histopathological changes observed in 14-day and/or 28-day repeated-dose studies – genotoxic carcinogens but not liver targeted

We evaluated 6 Group B chemicals (Fig.3). The response frequencies for these chemicals were liver micronucleus induction (33% [2/6]), hypertrophy (33% [2/6]), tissue injuries (17% [1/6]), regenerative changes (17% [1/6]), and inflammatory changes (17% [1/6]).

Group B chemicals did not cause proliferation of oval cells or bile duct epithelial cells, focus of altered hepatocytes, non-regenerative or regenerative hyperplasia, adenoma, or liver cancer. MNNG showed 3 responses, but 3 chemicals (MMC, MMS, and KA) showed only one or two responses. CP and KBrO_3_ did not show any targeted responses.

## Discussion

Few Group A chemicals caused the focus of altered hepatocytes (21% [3/14]) or adenoma (7% [1/14]). No chemical Group A or B exhibited non-regenerative or regenerative hyperplasia or liver cancer. The latter parameters are recognized as the most credible indicators of hepatocarcinogenesis [46–48]. The present study was a retrospective survey of short-term study with 14- or 28-day repeated dose design, and such findings are not expected. Thus, we selected 6 responses that expected to occur very early in the process of carcinogenesis, including hypertrophy, proliferation of oval cells or bile duct epithelial cells, tissue injuries, mutation (including liver micronucleus induction), regenerative changes, and inflammatory changes (Table 2).

**Table 2:**
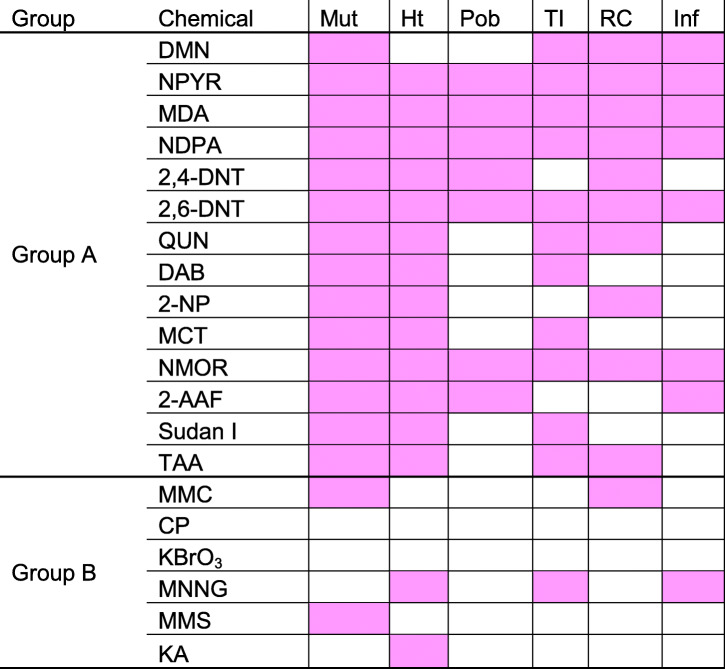
Histopathological changes and induction of liver micronuclei seen as very early responses of hepatocarcinogenesis

Group A: Genotoxic hepatocarcinogens, Group B: Genotoxic carcinogens but not liver targeted

Mut: Liver MN induction, Ht: Hypertrophy, Pob: Proliferation oval cell or bile duct, TI: Tissue injuries, RC: Regenerative change, Inf: Inflammatory

All 14 Group A chemicals were positive for liver micronucleus assay; only 2 of 6 Group B chemicals induced micronuclei. These 2 chemicals, namely, MMC and MMS, are carcinogens but are not liver-targeted. Both are direct-acting genotoxic chemicals that are used as positive controls in genotoxicity tests and induce micronuclei in various tissues, including the liver [2]. Therefore, liver micronucleus induction was considered to be a useful indicator for possible hepatocarcinogenesis. Speculatively, the chromothripsis could involve fragmentation and subsequent reassembly of a single chromatid from a micronucleus [49, 50]. Chromothripsis is a new concept for mutational process; it involves genome reorganization associated with micronuclei. This process might elucidate the mechanisms for the production of micronuclei and genome instability and cellular evolution essential in complex diseases such as cancer [50].

In addition to liver micronucleus induction, many Group A chemicals exhibited two or more of the other five responses assumed to be early predictors of carcinogenesis. Contrarily, in Group B chemicals, liver micronucleus induction was not observed in 4 out of 6 chemicals. These chemicals demonstrated varying responses, including hypertrophy (50% [2/4]), proliferation oval cells or bile duct epithelial cells (0% [0/4]), tissue injuries (25% [1/4]), regenerative changes (0% [0/4]), and inflammatory changes (25% [1/4]). Further, hypertrophy, proliferation oval cells or bile duct, tissue injuries, and inflammatory were not observed in the two chemicals that were positive for liver micronucleus induction. Only regenerative changes were observed for one of these chemicals. Thus, even if a chemical is found to be positive for liver micronucleus induction, negative results for all other pathological findings indicative of early stages of carcinogenesis suggest a low probability of cancer development in the liver.

Much debate has occurred over the issue of whether hypertrophy is a key early response in hepatotoxicity or hepatocarcinogenicity in rodent toxicity studies [51–54].

We suggest that hypertrophy in the liver without micronucleus induction does not predict future hepatocarcinogenesis. Hypertrophy with micronucleus induction is, however, closely related to hepatocarcinogenesis. Clofibrate is a typical non-genotoxic hepatocarcinogen that induces hepatocyte hypertrophy and liver micronuclei [2].

A recently developed formalin fixation method for the liver micronucleus assay [21, 55] enables retrospective evaluation using formalin-fixed liver samples from general toxicity and carcinogenicity studies completed in the past. With this method, the prediction of hepatocarcinogenicity of a test substance with accuracy is possible using data from 2- and 4-week repeated-dose toxicity studies, including previously published work.

## Conclusion

Liver micronucleus induction can be employed to predict hepatocarcinogenesis. The combination of this assay with histopathological findings observed in the early stages of the carcinogenic process (hypertrophy, proliferation of oval cells or bile duct epithelial cells, tissue injuries, regenerative changes, and inflammatory changes) can increase the accuracy of the prediction even in a short-term repeated dose study of 2 or 4 weeks.

## Data Availability

All data generated or analyzed during this study are included in this published article.

## Abbreviations

DMN: Dimethylnitrosamine
NPYR: *N*-Nitrosopyrrolidine
MDA: 4,4′-Methylenedianiline
NDPA: *N*-Nitrosodipropylamine
2,4-DNT: 2,4-Dinitrotoluene
2,6-DNT: 2,6-Dinitrotoluene
QUN: Quinoline
DAB: *p*-Dimethylaminoazobenzene
2-NP: 2-Nitropropane
MCT: Monocrotaline
NMOR: *N*-Nitrosomorpholine
2-AAF: 2-Acetylaminofluorene
Sudan I: C.I.solvent yellow 14
TAA: Thioacetamide
MMC: Mitomycin C
CP: Cyclophosphamide H_2_O
KBrO_3_: Potassium bromate
MNNG: *N*-Methyl-*N*′-nitro-*N*-nitrosoguanidine
MMS: Methyl methanesulfonate
KA: Kojic acid
ICH: The International Council for Harmonisation of Technical Requirements for Pharmaceuticals for Human Use
IARC: International Agency for Research on Cancer
JEMS: The Japanese Environmental Mutagen Society
MMS: Mammalian Mutagenicity Study Group
CSGMT: The Collaborative Study Group for the Micronucleus Test

## References

[1] Fukushima S (2008). Medium-term tests for carcinogens and carcinogenic hazard evaluation for humans. Journal of Occupational Safety and Health.

[2] Hamada S, Ohyama W, Takashima R, Shimada K, Matsumoto K, Kawakami S, Uno F, Sui H, Shimada Y, Imamura T, Matsumura S, Sanada H, Inoue K, Muto S, Ogawa I, Hayashi A, Takayanagi T, Ogiwara Y, Maeda A, Okada E, Terashima Y, Takasawa H, Narumi K, Wako Y, Kawasako K, Sano M, Ohashi N, Morita T, Kojima H, Honma M, Hayashi M (2015). Evaluation of the repeated-dose liver and gastrointestinal tract micronucleus assays with 22 chemicals using young adult rats: summary of the collaborative study by the Collaborative Study Group for the Micronucleus Test (CSGMT)/The Japanese Environmental Mutagen Society (JEMS) - Mammalian Mutagenicity Study Group (MMS). Mutat Res.

[3] Natarajan AT, Tates AD, Van Buul PP, Meijers M, De Vogel N (1976). Cytogenetic effects of mutagens/carcinogens after activation in a microsomal system in vitro I. induction of chromosome aberrations and sister chromatid exchanges by diethylnitrosamine (DEN) and dimethylnitrosamine (DMN) in CHO cells in the presence of rat-liver microsomes. Mutat Res.

[4] Ashby J, Tennant RW (1991). Definitive relationships among chemical structure, carcinogenicity and mutagenicity for 301 chemicals tested by the U. S NTP Mutat Res.

[5] Morita T, Asano N, Awogi T, Sasaki YF, Sato S, Shimada S, Sutou S, Suzuki T, Wakata A, Sofuni T, Hayashi M (1997). Evaluation of the rodent micronucleus assay in the screening of IARC carcinogens (group 1, 2A and 2B). The summary report of the 6th collaborative study by CSGMT/JEMS∙MMS. Mutat Res.

[6] George E, Westmoreland C (1991). Evaluation of the in vivo genotoxicity of the structural analogues 2,6-diaminotoluene using the rat micronucleus test and rat liver UDS assay. Carcinogenesis..

[7] Tates AD, Neuteboom I, Hofker M, Den Engelse L (1980). A micronucleus technique for detecting clastogenic effects of mutagens/carcinogens (DEN, DMN) in hepatocytes of rat liver in vivo. Mutat Res.

[8] Tates AD, Den Engelse L (1989). The role of short-lived lesions in the induction of micronuclei in rat liver by ethylnitrosourea and methyl methanesulfonate: the importance of experimental design. Mutat Res.

[9] Angelosanto FA (1995). Tissues other than bone marrow that can be used for cytogenetic analysis. Environ Mol Mutagen.

[10] Braithwaite I, Ashby J (1988). A non-invasive micronucleus assay in the rat liver. Mutat Res.

[11] Ashby J, Lefevre PA (1989). The rat-liver carcinogen N-nitrosomorpholine initiates unscheduled DNA synthesis and induces micronuclei in the rat liver in vivo. Mutat Res.

[12] Suzuki H, Ikeda N, Kobayashi K, Terashima Y, Shimada Y, Suzuki T, Hagiwara T, Hatakeyama S, Nagaoka K, Yoshida J, Saito Y, Tanaka J, Hayashi M (2005). Evaluation of liver and peripheral blood micronucleus assays with 9 chemicals using young rats. A study by the collaborative study Group for the Micronucleus Test (CSGMT)/Japanese environmental mutagen society (JEMS)-mammalian mutagenicity study group (MMS). Mutat Res.

[13] Suzuki H, Takasawa H, Kobayashi K, Terashima Y, Shimada Y, Ogawa I, Tanaka J, Imamura T, Miyazaki A, Hayashi M (2009). Evaluation of a liver micronucleus assay with 12 chemicals using young rats (II): a study by the collaborative study Group for the Micronucleus Test/Japanese environmental mutagen society-mammalian mutagenicity study group. Mutagenesis..

[14] Takasawa H, Suzuki H, Ogawa I, Shimada Y, Kobayashi K, Terashima Y, Matsumoto H, Aruga C, Oshida K, Ohta R, Imamura T, Miyazaki A, Kawabata M, Minowa S, Hayashi M (2010). Evaluation of a liver micronucleus assay in young rats (III): a study using nine hepatotoxicants by the collaborative study Group for the Micronucleus Test (CSGMT)/Japanese environmental mutagen society (JEMS)-mammalian mutagenicity study group (MMS). Mutat Res.

[15] Takasawa H, Suzuki H, Ogawa I, Shimada Y, Kobayashi K, Terashima Y, Matsumoto H, Oshida K, Ohta R, Imamura T, Miyazaki A, Kawabata M, Minowa S, Maeda A, Hayashi M (2010). Evaluation of a liver micronucleus assay in young rats (IV): a study using a double-dosing/single-sampling method by the collaborative study Group for the Micronucleus Test (CSGMT)/Japanese environmental mutagen society (JEMS)-mammalian mutagenicity study group (MMS). Mutat Res.

[16] Rossi AM, Romano M, Zaccaro L, Pulci R, Salmona M (1987). DNA synthesis, mitotic indices, drug-metabolising systems and cytogenetic analyses in regenerating rat liver. Mutat Res.

[17] Parton JW, Garriott ML (1997). An evaluation of micronucleus induction in bone marrow and in hepatocytes isolated from collagenase perfused liver or from formalin-fixed liver using four-week-old rats treated with known clastogens. Environ Mol Mutagen.

[18] Kato R, Yamazoe Y. Sex-specific cytochrome P450 as a cause of sex and species-related differences in drug toxicity. Toxicol Lett. 1992;64/65:661–7. 10.1016/0378-4274(92)90245-f 1471220

[19] Narumi K, Ashizawa K, Takashima R, Takasawa H, Katayama S, Tsuzuki Y, Teramoto H, Morita T, Hayashi M, Hamada S (2012). Development of repeated-dose liver micronucleus assay using adult rats: an investigation of diethylnitrosamine and 2, 4-diaminotoluene. Mutat Res.

[20] ICH HARMONISED GUIDELINE. S1B(R1), ADDENDUM TO THE GUIDELINE ON TESTING FOR CARCINOGENICITY OF PHARMACEUTICALS, draft version, endorsed on 10 may 2021. Currently under public consultation.

[21] Hamada S, Shigano M, Kawakami S, Ueda M, Sui H, Yamada K, Hagio S, Momonami A, Maeda A, Terashima Y, Ohyama W, Morita T, Hayashi M (2019). Evaluation of the novel liver micronucleus assay using formalin-fixed tissues. Genes Environ.

[22] L S. Gold, The carcinogenic potency database (CPDB). 2011. https://files.toxplanet.com/cpdb/indices.html.

[23] Greenblatt M, Lijinsky W (1972). Nitrosamine studies: neoplasms of liver and genital mesothelium N-nitrosopyrrolidine-treated MRC rats. J Natl Cancer Inst.

[24] IARC, IARC Monographs on the evaluation of the carcinogenic risk of chemicals to humans. Some Chemicals Used in Plastic and Elastomers 1986;39:347–365. 3465693

[25] Hirao K, Shinohara Y, Tsuda H, Fukushima S, Takahashi M, Ito N (1976). Carcinogenic activity of quinoline on rat liver. Cancer Res.

[26] IARC, IARC Monographs on the evaluation of the carcinogenic risk of chemicals to humans, Re-evaluation of Some Organic Chemicals Hydrazine and Hydrogen Peroxide 1999;71:1059–1078. PMC7681305 10507919

[27] IARC, IARC Monographs on the evaluation of the carcinogenic risk of chemicals to humans, A Review of Human Carcinogens: Pharmaceuticals 2012;100A: 63–90.

[28] Takashima R, Takasawa H, Kawasako K, Ohyama W, Okada E, Narumi K, Fujiishi Y, Wako Y, Yasunaga K, Hattori A, Kawabata M, Nakadate K, Nakagawa M, Hamada S (2015). Evaluation of a repeated dose liver micronucleus assay in rats treated with two genotoxic hepatocarcinogens, dimethylnitrosamine and 2-acetylaminofluorene: the possibility of integrating micronucleus tests with multiple tissues into a repeated dose general toxicity study. Mutat Res.

[29] Ogawa I, Hagio S, Furukawa S, Abe M, Kuroda Y, Hayashi S, Wako Y, Kawasako K (2015). Evaluation of repeated dose micronucleus assays of the liver using *N*-nitrosopyrrolidine: a report of the collaborative study by CSGMT/JEMS MMS. Mutat Res.

[30] Terashima Y, Yokoi R, Takakura I, Saitou E, Wako Y, Kawasako K, Souma S, Tamura T (2015). Detection of micronuclei in hepatocytes isolated from young adult rats repeatedly treated with *N*-nitrosodi-*n*-propylamine. Mutat Res.

[31] Maeda A, Tsuchiyama H, Asaoka Y, Hirakata M, Miyoshi T, Oshida K, Miyamoto Y (2015). Evaluation of the repeated-dose liver micronucleus assay using 2,4-dinitrotoluene: a report of a collaborative study by CSGMT/JEMS MMS. Mutat Res.

[32] Uno F, Tanaka J, Ueda M, Nagai M, Fukumuro M, Natsume M, Oba M, Akahori A, Masumori S, Takami S, Wako Y, Kawasako K, Kougo Y, Ohyama W, Narumi K, Fujiishi Y, Okada E, Hayashi M (2015). Repeated-dose liver and gastrointestinal tract micronucleus assays for quinoline in rats. Mutat Res.

[33] Shimada Y, Sui H, Wako Y, Kawasako K (2015). The evaluation of the repeated-dose liver micronucleus assay with *p*-dimethylaminoazobenzene. Mutat Res.

[34] Kawakami S, Araki T, Nakajima M, Kusuoka O, Uchida K, Sato N, Tanabe Y, Takahashi K, Wako Y, Kawasako K, Tsurui K (2015). Repeated-dose liver micronucleus assay: an investigation with 2-nitropropane, a hepatocarcinogen. Mutat Res.

[35] Takashima R, Takasawa H, Wako Y, Kawasako K, Yasunaga K, Hattori A, Kawabata M, Nakadate K, Nakagawa M, Hamada S (2015). Micronucleus induction in rat liver and bone marrow by acute vs. repeat doses of the genotoxic hepatocarcinogen monocrotaline. Mutat Res.

[36] Hayashi A, Kosaka M, Kimura A, Wako Y, Kawasako K, Hamada S (2015). Evaluation of the repeated-dose liver micronucleus assay using *N*-nitrosomorpholine in young adult rats: report on collaborative study by the collaborative study Group for the Micronucleus Test (CSGMT)/Japanese environmental mutagen society (JEMS) – mammalian mutagenicity study (MMS) group. Mutat Res.

[37] Matsumura S, Ikeda N, Hamada S, Ohyama W, Wako Y, Kawasako K, Kasamatsu T, Nishiyama N (2015). Repeated-dose liver and gastrointestinal tract micronucleus assays with CI solvent yellow 14 (Sudan I) using young adult rats. Mutat Res.

[38] Sui H, Matsumoto H, Wako Y, Kawasako K (2015). Evaluation of in vivo genotoxicity by thioacetamide in a 28-day repeated-dose liver micronucleus assay using male young adult rats. Mutat Res.

[39] Matsumoto K, Zaizen K, Miyamoto A, Wako Y, Kawasako K, Ishida H (2015). Evaluation of the repeated dose liver micronucleus assay using young adult rats with cyclophosphamide monohydrate: a report of a collaborative study by CSGMT/JEMS MMS. Mutat Res.

[40] Okada E, Fujiishi Y, Narumi K, Kado S, Wako Y, Kawasako K, Kaneko K, Ohyama W (2015). Evaluation of repeated dose micronucleus assays of the liver and gastrointestinal tract using potassium bromate: a report of the collaborative study by CSGMT/JEMS MMS. Mutat Res.

[41] Takayanagi T, Wako Y, Kawasako K, Hori H, Fujii W, Ohyama W. Repeated dose liver and gastrointestinal tract micronucleus assays using *N*-methyl-*N*’-nitro-Nnitrosoguanidine in young adult rats. Mutat Res. 2015;780–781:100–6. 10.1016/j.mrgentox.2014.12.009 25892628

[42] Muto S, Yamada K, Kato T, Wako Y, Kawasako K, Iwase Y, Uno Y. Assessment of methyl methanesulfonate using the repeated-dose liver micronucleus assay in young adult rats. Mutat Res. 2015;780–781:107–10. 10.1016/j.mrgentox.2014.08.008 25892629

[43] Takayanagi T, Takashima R, Wako Y, Kawasako K, Tanaka Y, Hori H, Fujii W. Repeated dose liver micronucleus assay using clofibrate in young adult rats. Mutat Res. 2015;780–781:117–22. 10.1016/j.mrgentox.2015.01.002 25892631

[44] Inoue K, Ochi A, Koda A, Wako Y, Kawasako K, Doi T (2015). The 14-day repeated dose liver micronucleus test with methapyrilene hydrochloride using young adult rats. Mutat Res.

[45] Ogawa K (2009). Molecular pathology of early stage chemically induced hepatocarcinogenesis. Pathol Int.

[46] Sugitani S, Sakamoto M, Ichida T, Genda T, Asakura H, Hirohashi S (1998). Hyperplastic foci reflect the risk of multicentric development of human hepatocellular carcinoma. J Hepatol.

[47] Farber E (1956). Similarities in the sequence of early histological changes induced in the liver of the rat by ethionine, 2-acetylamino-fluorene, and 3′-methyl-4-dimethylaminoazobenzene. Cancer Res.

[48] Laconi S, Pani P, Pillai S, Sarma DSR, Laconi E (2001). A growth-constrained environment drives tumor progression in vivo. Proc Natl Acad Sci U S A.

[49] Zhang CZ, Spektor A, Cornils H, Francis JM, Jackson EK, Liu S, Meyerson M, Pellman D (2015). Chromothripsis from DNA damage in micronuclei. Nature.

[50] Ye CJ, Sharpe Z, Alemara S, Mackenzie S, Liu G, Abdallah B, Horne S, Regan S, Heng HH (2019). Micronuclei and genome chaos: changing the system inheritance. Genes.

[51] Hall AP, Elcombe CR, Foster JR, Harada T, Kaufmann TW, Knippel A, Küttler K, Malarkey DE, Maronpot RR, Nishikawa A, Nolte T, Schulte A, Strauss V, York MJ (2012). Liver hypertrophy: a review of adaptive (adverse and non-adverse) changes —conclusions from the 3rd international ESTP expert workshop. Toxicol Pathol.

[52] Robert RM, Yoshizawa K, Nyska A, Harada T, Flake G, Mueller G, Singh B, Ward JM (2010). Hepatic enzyme induction: histopathology. Toxicol Pathol.

[53] Elcombe CR, Peffer RC, Wolf DC, Bailey J, Bars R, Bell D, Cattley RC, Ferguson SS, Geter D, Goetz A, Goodman JI, Hester S, Jacobs A, Omiecinski CJ, Schoeny R, Xie W, Lake BG (2014). Mode of action and human relevance analysis for nuclear receptor-mediated liver toxicity: a case study with phenobarbital as a model constitutive androstane receptor (CAR) activator. Crit Rev Toxicol.

[54] Rusyn I, Peters JM, Cunningham ML (2006). Modes of action and species-specific effects of di-(2-ethylhexyl)phthalate in the liver. Effects of DEHP in the Liver: Modes of Action and Species-Specific Differences Crit Rev Toxicol.

[55] Shigano M, Takashima R, Takasawa H, Hamada S (2016). Optimization of specimen preparation from formalin-fixed liver tissues for liver micronucleus assays: hepatocyte staining with fluorescent dyes. Mutat Res.

